# Ultrasonic driven resveratrol induced egg-derived amyloid-like fibrils hydrocolloids for stabilizing aqueous cyanidin-3-O-glucoside

**DOI:** 10.1016/j.ultsonch.2026.107767

**Published:** 2026-02-08

**Authors:** Xuke Han, Chan Zhu, Yifeng Shen, Minmin Ai, Lue Ha

**Affiliations:** a Key Laboratory of Acupuncture and Medicine in Shaanxi Province, College of Acupuncture & Tuina, Shaanxi University of Chinese Medicine, Xianyang 712046, China; b Shaanxi TCM Diagnosis and Treatment Technology and Equipment R&D Collaborative Innovation Center, Shaanxi University of Chinese Medicine, Xianyang 712046, China; c Key Laboratory of Birth Defects and Related Gynecological Diseases, Traditional Chinese Medicine Department, West China Second Hospital of Sichuan University, Chengdu 610021, China; d TCM Regulating Metabolic Diseases Key Laboratory of Sichuan Province, Hospital of Chengdu University of Traditional Chinese Medicine, Chengdu 610032, China; e College of Food Science, South China Agricultural University, Guangzhou 510642, China

**Keywords:** Amyloid fibrils, Resveratrol, Hydrocolloids, Emulsion-hydrocolloids, Cyanidin-3-O-glucoside

## Abstract

This study investigated a low-frequency ultrasound-driven, resveratrol-induced hydrocolloid composed of ovalbumin and lysozyme amyloid-like fibrils for the efficient stabilization of aqueous cyanidin-3-O-glucoside. Results demonstrated that resveratrol, under ultrasonic treatment, acted as a molecular bridge, disrupting the long-range order of linear *β*-sheets and promoting fibril cross-linking via intramolecular hydrogen bonds. This process significantly reduced the average particle size of the fibrils, transforming the system into a three-dimensional hydrocolloid network with improved viscoelasticity. The resulting hydrocolloid with adjusted surface hydrophobicity and surface hydrophobicity effectively stabilized emulsion- hydrocolloids loaded with cyanidin-3-O-glucoside, yielding uniform droplets with enhanced stability against aggregation and oxidation during storage. The confidence interval of the Cole-Cole curve of the emulsion hydrocolloid narrated and linearized with the increase in resveratrol ratio, and the formed single relaxation network microstructure significantly enhanced the environmental stimulation stability of the emulsion hydrocolloid. *In vitro* digestion studies revealed that prepared emulsion-hydrocolloids significantly enhanced the bioaccessibility of cyanidin-3-O-glucoside. Furthermore, in a *Caco-2* cell monolayer model, the digestively derived micelles from the emulsion-hydrocolloid notably improved the absorption rate of cyanidin-3-O-glucoside over 120 min and exhibited strong cytoprotective effects under H_2_O_2_-induced oxidative stress by effectively reducing intracellular ROS levels, increasing SOD activity, and decreasing MDA content. All treatments maintained high cell viability (>95%), indicating excellent biocompatibility. These findings suggest that the ultrasound-assisted resveratrol-induced fibril hydrocolloid is a highly promising vehicle for improving the stability, bioavailability, and bioactivity of hydrophilic polyphenols.

## Graphical abstract

**Figure:**
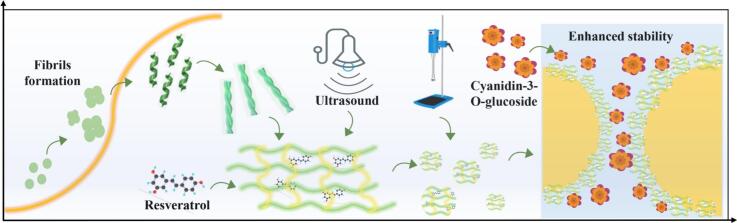

## 1 Introduction

Amyloid fibrils offer a novel material option for the design of natural protein-based hydrocolloids. Certain egg-derived proteins, such as ovalbumen fibrils (OVAf) and lysozyme fibrils (LYZf), form fibrous assemblies upon induction by extreme acid and heat treatment[1]. These assemblies feature continuous *β*-sheets as the molecular core and exhibit a fibrous morphology with a high aspect ratio at the macroscale[2]. They not only possess excellent mechanical strength and thermal stability but also allow precise regulation of surface charge and hydrophobicity by modulating assembly conditions. OVAf and LYZf demonstrate inherent structural complementarity. OVAf forms rigid long fibrils that provide spatial skeletal support, while LYZf exhibits a short fibrous branched structure due to its high charge density[3]. However, OVAf tends to undergo random aggregation via hydrophobic interactions due to its fibril rigidity, forming loose aggregates rather than a continuous network [4]. In contrast, LYZf can only maintain a low-viscosity solution state due to insufficient intermolecular entanglement, failing to provide adequate elastic modulus for forming a stable hydrocolloid system[5]. Therefore, the key technical challenge in constructing high-performance amyloid fibril hydrocolloids lies in overcoming the random aggregation of OVAf and insufficient entanglement of LYZf through external interventions, thereby guiding their directional and efficient synergistic assembly instead of simple physical mixing.

Resveratrol (RES) is a natural polyphenolic compound with potent antioxidant activity[6]. Previous studies have reported that self-assembled hydrocolloids can be formed by introducing RES into OVAf and adjusting the pH to 6.5[7]. The phenolic hydroxyl groups of RES form hydrogen bond networks with amino groups on fibril surfaces, while its aromatic rings insert into the *β*-sheet core to enhance rigidity[8], [9]. This overcomes electrostatic repulsion to enable hierarchical assembly and alters the fibril assembly pathway through *π-π* stacking interactions, inducing a transition of linear protein fibrils from disordered aggregation to branched cross-linking and promoting phase transition of the system[10]. This hydrocolloid network possesses both certain mechanical strength and dynamic responsiveness, making it favorable for stabilizing sensitive bioactive substances[11]. C3G, a representative anthocyanin widely present in berries, exhibits low retention rates due to the high reactivity of its phenolic hydroxyl groups[12]. Its strong hydrophilicity results in hydrolysis into glycosides in the upper small intestine after oral administration, leading to low bioavailability[13]. This dilemma of high activity, low stability, and low bioavailability constitutes a bottleneck for the industrial application of cyanidin-3-O-glucoside (C3G) [14]. There is an urgent need to develop novel delivery carriers integrating stable protection, controlled release, and efficient absorption functions.

Low-frequency ultrasound technology has demonstrated significant application potential in the field of food protein assembly and modification due to its unique physical effects[15]. This technology influences biomacromolecules primarily through cavitation effects and mechanical vibration. Compared with high-frequency ultrasound, low-frequency ultrasound exhibits stronger penetration ability and deeper tissue propagation, enabling its energy to act more effectively on protein solutions or suspension systems, thereby promoting the unfolding, orientation, and interaction of molecular chains[16]. Studies have shown that low-frequency ultrasound can effectively regulate protein assembly kinetics, such as inducing the formation of fibrous structures, improving the homogeneity of hydrocolloid networks, and facilitating interactions between functional components and proteins, thus optimizing the construction of delivery systems[17], [18]. Therefore, the use of low-frequency ultrasound as a physical field-assisted tool provides a novel strategy for developing advanced food colloids and delivery systems based on protein self-assembly.

Thus, to complement the inherent deficiencies of OVAf and LYZf, the present study aims to construct a low-frequency ultrasound-driven, RES-induced OVAf-LYZf composite hydrocolloid for stabilizing C3G in the aqueous phase. The study will focus on investigating the regulatory mechanism of RES-induced fibril gelation under ultrasound fields, the loading and release behavior of C3G from the hydrocolloid, its digestive stability and bioaccessibility in simulated gastrointestinal environments, as well as its protective effect against oxidative stress in intestinal epithelial cells.

## 2 Materials and methods

### 2.1 Materials

OVA (ovalbumin, ≥90%) and LYZ (lysozyme, from chicken egg white, ≥95%) were purchased from Yuanye Bio-technology Co., Ltd (Shanghai, China). Nile Red, 8-aniline-1-naphthalenesulfonic acid (ANS), fluorescein 5-isothiocyanate (FITC), pepsin (from porcine gastric mucosa, 3256 U/mg), and pancreatin (from porcine pancreas, 1587 U/mg) were purchased from Sigma-Aldrich (St. Louis, MO, USA). Bile salts (porcine), Calcein-AM, and propidium iodide (PI) were obtained from Macklin Biochemical Co., Ltd (Shanghai, China). All other chemicals were of analytical grade.

### 2.2 Preparation of OVAf and LYZf

LYZf was prepared according to a previous method with modifications[19]. Briefly, lysozyme (0.4 g) was dissolved in 20 mL of deionized water to obtain a 2.0% (w/v) solution. The pH of the solution was adjusted to 2.0 using 2 M HCl. After stirring at 300 rpm for 1 h, the solution was heated at 90 °C for 24 h in a water bath shaker (100 rpm). Subsequently, the solution was immediately cooled in an ice-water bath for 30 min and stored at 4 °C. Similarly, OVA (0.8 g) was dissolved in 10 mL of deionized water to form a 4.0% (w/v) solution. After hydration at 4 °C for 12 h, the pH was adjusted to 2.0, followed by heating at 90 °C for 24 h. The resulting fibril dispersion was cooled and diluted to a final concentration of 4 mg/mL with deionized water.

### 2.3 Preparation of ultrasonic-driven RES-induced fibrils hydrocolloids

RES was dissolved in a 70% (v/v) ethanol solution to prepare stock solutions. The pre-prepared LYZf dispersion (1 mL) was mixed with an equal volume of the OVAf dispersion (4 mg/mL). Subsequently, 0.2 mL of RES stock solution was added to the protein mixture to achieve final RES concentrations of 0, 50, 100, and 200 μM. The mixtures were vortexed thoroughly and then treated with low-frequency ultrasound (VSX800, Sonics, America) at 100 w, 20 kHz and an amplitude of 30% for 30 min, then incubated at 4 °C for 24 h to facilitate hydrocolloid formation. A portion of the hydrocolloid samples was freeze-dried for further characterization.

### 2.4 Characterization of RES induced OVAf and LYZf hydrocolloids

The particle size, polydispersity index (PDI), and ζ-potential of the hydrocolloid dispersions (diluted to 1 mg/mL protein concentration) were measured using a ZetaSizer Nano ZS90 instrument (Malvern Instruments, UK). Measurements were performed at 25 °C after 120 s of equilibration. Steady-state viscosity measurements were conducted using an Anton Paar MCR 301 rheometer (Graz, Austria) equipped with a cone-plate geometry (CP50). Shear rate sweeps were performed from 0.1 to 100 s^−1^ at 25 °C with Power-law fitting (y = *a*γ^b^, y represents viscosity (Pa·s), *a* is the viscosity index (Pa·sn), *γ* is the shear rate (s^−1^), and *b* is the unitary power-law index), frequency sweeps were conducted from 1-10 rad/s with 1% strain. For atomic force microscopy (AFM), samples were diluted to 5 ng/mL. An 8 µL aliquot was deposited onto a freshly cleaved mica surface and air-dried for 12 h in a desiccator. Imaging was performed using a Dimension FastScan AFM (Bruker, USA) in tapping mode.

Intrinsic fluorescence spectra were recorded using an RF5301-PC (Shimadzu, Japan). Protein solutions (1 mg/mL) were excited at 295 nm, and emission spectra were collected from 300 to 420 nm. Thioflavin T (ThT) fluorescence assays were performed by mixing 40 μL of sample (4 mg/mL) with 4 mL of ThT working solution (prepared in 10 mM phosphate buffer, pH 7.0). After 10 min of reaction, fluorescence was measured with excitation at 440 nm and emission from 460 to 560 nm. Fourier transform infrared (FTIR) spectra of freeze-dried samples were acquired using a Vertex 70 spectrometer (Bruker, Germany) over the range of 4000–400 cm^−1^ at a resolution of 4 cm^−1^. X-ray diffraction (XRD) patterns were recorded using an Ultima IV diffractometer (Rigaku, Japan) with Cu Kα radiation, scanning from 5° to 60° (2θ) at a speed of 5°/min. Surface hydrophobicity was determined using ANS as a fluorescent probe. Samples at different concentrations (0.1–4 mg/mL) were mixed with 20 μL of 8 mM ANS solution. Fluorescence intensity was measured at 470 nm (excitation 390 nm), and the initial slope of the fluorescence intensity versus protein concentration plot was calculated. Interfacial tension at the oil–water interface was measured using an optical contact angle meter (OCA-25, DataPhysics, Germany) via the pendant drop method. A droplet of hydrocolloid dispersion (0.2 mg/mL) was formed in corn oil, and the interfacial tension was monitored over time, and changes in interfacial tension were recorded.

### 2.5 Preparation of emulsion stabilized by fibrils hydrocolloids

The pre-formed hydrocolloid was first uniformly dispersed in the C3G solution (0.1%, w/v) under gentle stirring to achieve a final hydrocolloid concentration of 2% (w/v). Subsequently, corn oil was gradually added to the aqueous phase to attain an oil fraction of 50% (v/v). The mixture was then subjected to a two-step emulsification process: pre-emulsification​ was performed at 6,000 rpm for 1 min using a high-speed homogenizer, followed by high-speed homogenization​ at 12,000 rpm for an additional 2 min. The entire homogenization process was carried out in an ice-water bath to prevent thermal degradation. The final product was obtained as an emulsion hydrocolloids[20].

### 2.6 Characterization of emulsion- hydrocolloid stabilized by fibrils hydrocolloids

The droplet size distribution and specific surface area of the emulsion were determined by laser diffraction using a Mastersizer 3000 (Malvern Instruments, UK). For confocal laser scanning microscopy (CLSM), 20 μL of Nile Red solution (1 mg/mL in propylene glycol) was added to 1 mL of emulsion. After staining for 15 min, images were acquired using a TCS SP8 microscope (Leica, Germany) with excitation at 543 nm and emission collection at 633 nm. The rheological properties, including apparent viscosity (0.1 to 100 s^−1^) and frequency sweep (1–100 rad/s, 1% strain), of the emulsion were measured using the Anton Paar MCR 301 rheometer. Apparent viscosity was fitted with Cross model ($\eta =\eta_{\infty }+\frac{\eta_{0}-\eta_{\infty }}{1+{\left(\alpha \gamma \right)}^{n}}$, η_0_:Zero shear viscosity (Pa·s); η_∞_: Infinite shear viscosity (Pa·s); α: characteristic time (s); *n*: Flow index, degree of shear thinning), the frequency sweep results including G′, G″, G*, phase angle σ, the van Gurp-Palmen plot (phase angle δ vs. lgG*); (E) Cole-Cole plot (G'' vs. G'). The ionic strength (300 mM), heating (90℃) and storage (14 d) stability was evaluated by monitoring the change in droplet size of emulsions.

### 2.7 In-vitro simulated digestion

A simulated gastrointestinal tract (GIT) model was used[21]. The emulsion was first mixed with simulated salivary fluid (SSF, pH 6.8, containing 0.003 g/mL mucin) and incubated at 37 °C for 10 min. An equal volume of simulated gastric fluid (SGF, pH 2.0, containing 0.096 g pepsin, corresponding to a pepsin concentration of 60 U/mL) was then added, and the mixture was incubated for 60 min. Subsequently, simulated intestinal fluid (SIF, pH 7.0, containing trypsin at a concentration of 100 U/mL, lipase at 800 U/mL, and 0.5 wt% bile salts) was added, and the pH was maintained at 7.0 by automatic titration with 0.25 M NaOH for 2 h. The droplet size, ζ-potential, and microstructure of the emulsions were monitored after digestion. After intestinal digestion, the digest was ultracentrifuged at 40,000 × g for 30 min to collect the micelle fraction. The contents of C3G and RES in the micelles were quantified by HPLC. The sample was filtered through a 0.45 μm filter membrane and diluted with a methanol/water (50:50) solution. After the sample was filtered through a 0.45 μm filter membrane, it was diluted with methanol/water (50:50, v/v). Detection wavelengths: 306 nm and 520 nm; Chromatographic column: C18 column; Column temperature: 25 °C; Mobile phase A: 0.1% phosphoric acid aqueous solution; Mobile phase B: chromatographically pure acetonitrile; Flow rate: 0.8 mL/min. The injection volume was 5.0 μL. The running time was approximately 8 min[22]. Bioaccessibility was calculated as: (Amount of compound in micelles/Total amount of compound in initial emulsion) × 100%.

### 2.8 Caco-2 absorption rate of C3G in micelles

Caco-2 cells between passages 20 and 30 were seeded into 12-well Transwell inserts (pore size: 0.4 μm; diameter: 12 mm; growth area: 1.12 cm^2^) at a density of 2–3 × 10^5^ cells/mL. The apical (AP) compartment was filled with 0.5 mL of complete culture medium, while the basolateral (BL) compartment was supplemented with 1.5 mL of complete culture medium. The medium was refreshed every 48 h during the first week, followed by daily refreshing for two consecutive weeks thereafter. Successful monolayer formation was verified by measuring transepithelial electrical resistance (TEER) across the cell layer. A TEER value ≥ 300 Ω·cm^2^ was defined as a qualified intact monolayer. Micelles and Caco-2 cells were co-cultured for 24 h for cytotoxicity assay. After co-culture, the supernatant was discarded, and the cells were incubated with 100 µL of 10% (v/v) CCK-8 solution for 4 h. The absorbance of the samples at a wavelength of 450 nm was measured using a microplate reader. For live/dead cell staining, cells were incubated with 2 μM calcein-AM (AM) and 5 μg/mL PI, subsequent observation of Caco-2 cells was performed using a laser confocal scanning microscope. The excitation (Ex) and emission (Em) wavelengths were set as follows: Ex = 490 nm and Em = 515 nm for AM; Ex = 535 nm and Em = 617 nm for PI. For the absorption assay, pH 6.0 HBSS (500 μL) containing the safe-concentration micelle solution was added to the AP compartment, and pH 7.4 HBSS (1.5 mL) was added to the BL compartment. Samples were collected from the BL compartment at predetermined time points (0, 60, and 120 min). After each sampling, an equal volume of HBSS was replenished to the BL compartment to maintain a constant volume. The total concentrations of C3G and RES in the BL samples at each time point were quantified to calculate their respective absorption rates. Additionally, the concentration of C3G in the AP compartment, BL compartment, and intracellular fraction was determined separately.

### 2.9 Determination of antioxidant stress in Caco-2 cells by micelles

The cell density of Caco-2 was set at 2–3 × 10^5^ cells /mL. Subsequently, 100 μL of the cell suspension was inoculated into a 96-well plate for 24 h, and then 80 μL of fresh culture medium and 20 μL of micelles solution of different concentrations were added. Model group: The cell supernatant was treated with 100 μM H_2_O_2_ for 4 h, the supernatant was discarded, and then fresh culture medium was added for 24 h of culture. Sample group: The cell supernatant was treated with 100 μM H_2_O_2_ for 4 h, then the supernatant was discarded. Fresh culture medium and micelle solution were added, and then the cells were cultured for 24 h. Subsequently, the medium containing H_2_O_2_ was discarded and washed with PBS 2 to 3 times. Dilute the DCFH-DA probe in serum-free medium at a ratio of 1:1000 to a concentration of 5 μmol/ L. 100 μL of the staining solution was added to each well of the 96-well plate and incubated in a 37 °C, 5% CO_2_ incubator for 30 min. After incubation, wash the cells three times with serum-free medium to fully remove DCFH-DA that has entered the cells. Subsequently, a fluorescence inverted microscope (SP8, Leica, Germany) was used, with the channel set to FTIC to observe the intensity of the green fluorescence. The contents of SOD and MDA in cells were determined by using the kit, and the results were normalized to the total protein concentration.

### 2.10 Data analysis

All experiments were conducted at least in triplicate. The results are expressed as means accompanied by standard deviations (SDs). The test data were analyzed using the LSD test in IBM SPSS software (version 21.0), and multiple tests were conducted to determine the significance level, which was set at 5% (*P* < 0.05). All tests were repeated three times to avoid any randomness. The test data were plotted using Origin Pro 2021 software.

## 3 Results and discussion

### 3.1 Characterization the solution-sol-hydrocolloids transformation process of fibers

**Fig. 1:**
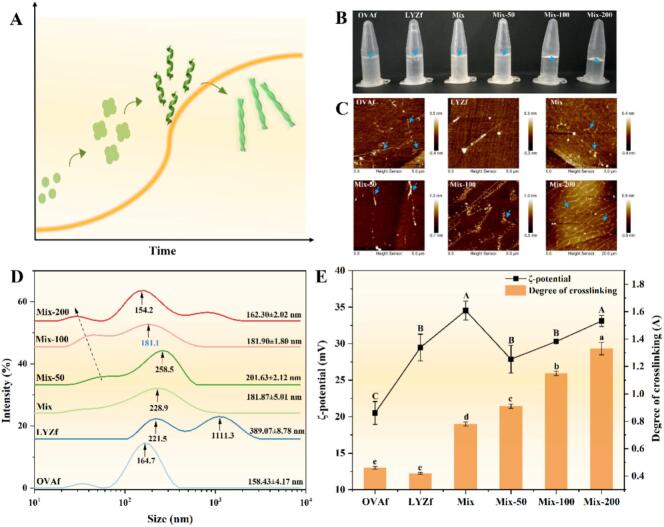
Characterization of resveratrol-induced ovalbumin and lysozyme nanofibril hydrocolloids: (A) Schematic diagram of amyloid fiber formation; (B) Visual appearance; (C) Atomic force microscope (AFM) images; (D) Size distribution; (E) Zeta potential and degree of crosslinking. Different lowercase letters above the bars indicate significant differences (*P* < 0.05).

Egg-derived proteins form amyloid fibrils under synergistic extreme acid and heat treatment (Fig. 1A). No hydrocolloids were formed without RES, whereas the mixed amyloid fibrils began to cross-link into hydrocolloids after adding 100–200 μM RES (Fig. 1B). Particle size distribution, average particle size, and ζ-potential of OVAf and LYZf were evaluated (Fig. 1D and 1E). The average particle size of OVAf was 158.43 ± 4.17 nm, showing a major peak at 164.7 nm. LYZf exhibited two distribution peaks at 1111.3 nm and 221.5 nm, respectively. After mixing, a single peak was observed with an average particle size of 181.87 ± 5.01 nm, indicating co-assembly, which was further confirmed by the degree of cross-linking (Fig. 1E). LYZf carried a strong positive charge, while OVAf had weak positivity; the two formed a homogeneous and stable complex through charge complementarity. Upon RES addition, OVAf and LYZf initiated cross-linking to form hydrocolloids, the main peak decreased from 258.5 nm to 154.2 nm, and the average particle size reduced from 201.63 ± 2.12 nm to 162.30 ± 2.02 nm (*P* < 0.05). The cavitation effect of low-frequency ultrasound, combined with the molecular bridging role of RES, effectively cleaved and restructured large fibrous aggregates into more homogeneous and smaller cross-linked units[8]. All samples exhibited positive ζ-potentials (Fig. 1E). The Mix and Mix-200 groups showed the highest absolute ζ-potential values (*P* < 0.05), while no significant difference was observed between the Mix-50, Mix-100, and LYZf groups (*P* > 0.05). OVAf had the lowest absolute ζ-potential, indicating that RES binding did not fully shield the positive charges on the fibril surfaces. Instead, it reduced electrostatic repulsion through local charge neutralization, promoting gelation. With increasing RES concentration, significant cross-linking of egg-derived fibrils occurred, leading to a marked increase in the degree of cross-linking (*P* < 0.05, Fig. 1E). RES disrupted the hydrophilic layer on the fibril surfaces, exposing the internal hydrophobic core, which enhanced hydrophobic interactions between fibrils and facilitated short-range cross-linking.

AFM observations revealed that OVAf and LYZf gradually formed typical amyloid fibrils (Fig. 1C). After mixing, some amyloid fibrils disappeared, and granular aggregates emerged. With the addition of RES, branched and interlocked cross-linked fibrous structures began to form: fibrils started to cross-link at 100 μM, and a 3D network was fully established at 200 μM. The energy provided by low-frequency ultrasound not only accelerated the diffusion and binding of RES molecules in the system but also generated strong shear forces that helped break initially loose aggregates, inducing fibril alignment and rearrangement. Charge shielding effects and competitive binding between the two types of fibrils disrupted their original structures. The polyphenolic structure of RES specifically bound to fibrils, stabilizing short fibril fragments and enabling fibril restructuring through intermolecular interactions[23]. At high concentrations, RES molecules may insert into the hydrophobic core of fibrils, inducing branched assembly and forming a 3D interlocked network. This process conforms to the percolation threshold theory, leading to the sol-hydrocolloids transition of the system.

**Fig. 2:**
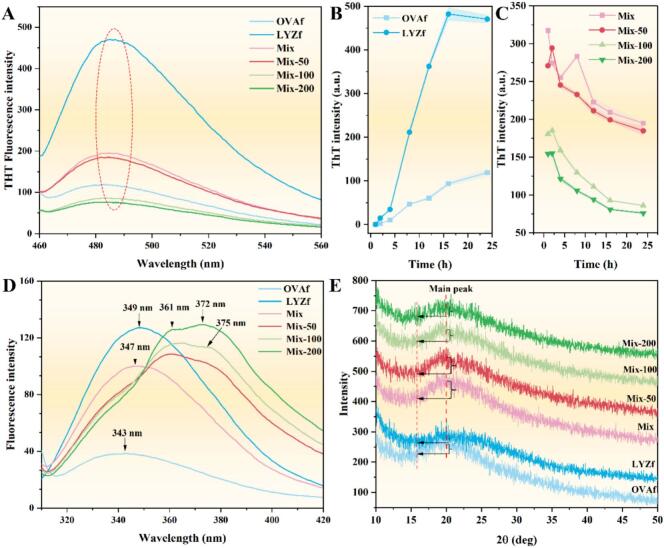
Spectroscopic and structural analysis of resveratrol-induced ovalbumin and lysozyme nanofibril hydrocolloids: (A) ThT fluorescence spectra after reaction for 24 h; (B) ThT fluorescence intensity during nanofibril formation; (C) ThT fluorescence intensity during hydrocolloid formation; (D) Intrinsic fluorescence spectra; (E) X-ray diffraction (XRD) patterns.

ThT fluorescence was used to characterize the degree of fibril formation (Fig. 2A–C). During acid-heat induction, ThT fluorescence intensity of LYZf and OVAf gradually increased, with LYZf exhibiting the strongest fluorescence (Fig. 2B), confirming that LYZ has a higher tendency to form amyloid fibrils than OVA. After mixing, the ThT fluorescence intensity was lower than that of LYZf over reaction time. RES addition further reduced ThT fluorescence, with the Mix-200 group showing the lowest intensity. RES significantly inhibited the formation of linear and regular amyloid fibrils in a concentration-dependent manner (Fig. 2C), consistent with AFM observations. Co-assembly of LYZf and OVAf resulted in partial dissociation of LYZf structures, while competitive binding between heterogeneous fibrils hindered the specific intercalation of ThT. RES formed a hydrogen bond network with the fibril backbone via its phenolic hydroxyl groups, directly competing for ThT binding sites[7]. Meanwhile, its hydrophobic aromatic ring structure could insert into the fibril hydrophobic core, disrupting the regular arrangement of *β*-sheets and significantly reducing the accessible surface area for ThT. RES regulated the fibril assembly pathway from linear extension to branched cross-linking, and the mechanical effects of low-frequency ultrasound promoted the entanglement and restructuring of the fibril network, driving the transition from solution to sol and eventually to hydrocolloids[8].

### 3.2 Structural and conformational changes

Changes in tertiary structure are shown in Fig. 2D. OVAf exhibited the lowest endogenous fluorescence intensity, while LYZf showed the highest. After equimolar mixing of the two fibrils, the fluorescence intensity decreased significantly. For mixed systems with RES, the fluorescence intensity increased with increasing RES concentration, and the wavelength corresponding to the peak shifted from 343 nm (OVAf), 349 nm (LYZf), and 347 nm (Mix group) to 361 nm. Additionally, the side peak at 375 nm increased markedly with rising RES concentration. Tryptophan residues in LYZf were located in a relatively hydrophobic microenvironment, whereas those in OVAf were more exposed. Heterogeneous fibril interactions increased the polarity of the tryptophan microenvironment. The aromatic rings of RES formed π-π stacking with the indole rings of tryptophan, constructing local hydrophobic microenvironments that offset fluorescence quenching caused by the hydrophilic environment[24]. The hydroxylated biphenyl structure of RES bound differently to both the hydrophobic core of fibrils and surface tryptophan residues, thereby inducing a dynamic conformational transition of proteins from β-sheet enrichment to a molten globule-like state[25]. Low-frequency ultrasound enhanced molecular collisions, enabling more RES molecules to wedge into the fibril interior effectively.

XRD results are presented in Fig. 2E. Diffraction peaks of all groups were concentrated around 20°. Compared with OVAf, LYZf exhibited a lower peak intensity with a rightward shift in peak position. After mixing OVAf and LYZf, the peak intensity increased, but it decreased with increasing RES concentration. All samples showed a characteristic peak of cross-*β*-sheet structure near ∼ 20°, reflecting the long-range ordered arrangement of *β*-sheets[26]. Upon mixing OVAf and LYZf, the two types of fibrils may have formed a more ordered heterogeneous lattice structure through complementary *β*-strand alignment or electrostatic interactions. RES disrupted the long-range order of linear *β*-sheets, an effect that may have been exacerbated by the cavitation effect of ultrasound. However, it promoted the formation and stabilization of short *β*-sheet fragments, which further cross-linked to form a nonlinear network, ultimately inducing an amorphous network structure and transforming rigid fibril crystals into a flexible cross-linked hydrocolloid network.

**Fig. 3:**
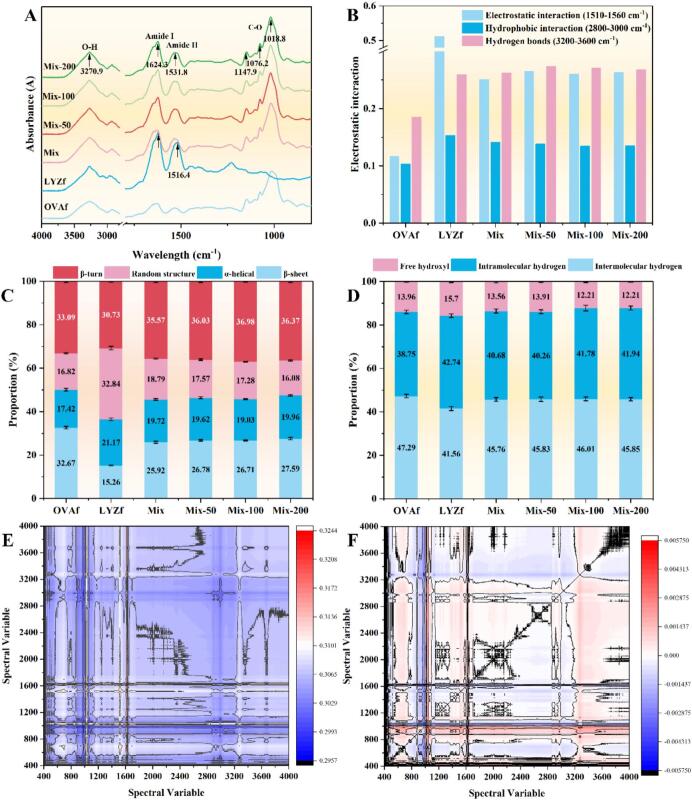
Analysis of molecular interactions and secondary structure of the nanofibril hydrocolloids: (A) FTIR spectra; (B) Proposed intermolecular interactions; (C) Secondary structure composition; (D) Hydrogen bonding analysis; (E) Synchronous and (F) asynchronous 2D-COS maps. Different lowercase letters above the bars indicate significant differences (*P* < 0.05).

FTIR results are displayed in Fig. 3A. The peak intensity at 1018 cm^−1^ was higher in the OVAf group than in the LYZf group, while LYZf did not show peaks at 1147, 1076, and 1018 cm^−1^, but exhibited significantly higher peak intensities at 1624 and 1531 cm^−1^ compared with OVAf. Peak changes in all groups were concentrated at 3270 cm^−1^; additionally, obvious vibrations were observed in Amide I, Amide II, and C-O groups, indicating that RES regulated fibril structure by affecting hydrogen bond networks and protein backbone vibrations[27]. Interactions in OVAf were weaker than those in LYZf and other groups. The LYZf group showed the highest absorbance for electrostatic interactions (1510–1560 cm^−1^), suggesting strong ionic bonds or salt bridges. In the mixed groups and RES-added groups, the absorbance for hydrophobic interactions (2800–3000 cm^−1^) decreased, while no significant changes were observed in electrostatic interactions and hydrogen bond interactions (3200–3600 cm^−1^; Fig. 3B) [28]. RES may preferentially regulate fibril assembly through insertion into the hydrophobic core rather than direct binding to polar groups, which is consistent with the results of crystal structure disruption and changes in the tryptophan microenvironment.

Secondary structure results are shown in Fig. 3C. LYZf had the lowest proportions of *β*-sheets and *β*-turns, while OVAf exhibited the lowest *α*-helix content (17.42%) and a significantly higher β-sheet content (32.67%) than LYZf (*P* < 0.05), indicating distinct structural characteristics of the two fibrils. After mixing OVAf and LYZf, the random coil content was significantly lower than that of LYZf alone (*P* < 0.05), while the *β*-sheet content increased slightly, suggesting that co-assembly of the two fibrils promoted the formation of more ordered *β*-sheet structures. With RES addition, the random coil content further decreased, and the *β*-sheet proportion increased to 27.59%, while no significant change was observed in the *α*-helix structure (*P* > 0.05). RES may optimize the molecular arrangement of the fibril network by selectively stabilizing *β*-sheet conformations and reducing disordered structures[29]. Combined with the aforementioned XRD results, it is speculated that RES induces the formation of a more flexible and stable fibrous hydrocolloid network by enhancing the stacking order of *β*-sheet.

Hydrogen bond type results are presented in Fig. 3D. OVAf had the lowest proportion of intramolecular hydrogen bonds (38.75%) and the highest proportion of intermolecular hydrogen bonds (47.29%), while the LYZf group showed the highest proportion of free hydroxyl groups (15.7%) and the lowest proportion of intermolecular hydrogen bonds (41.56%). After mixing and with increasing RES concentration, the proportion of free hydroxyl groups decreased from 13.56% to 12.21%, the proportion of intramolecular hydrogen bonds increased from 40.68% to 41.94%, and no significant change was observed in the proportion of intermolecular hydrogen bonds (*P* > 0.05), indicating reduced free hydroxyl groups, enhanced intramolecular hydrogen bonds, and stable intermolecular hydrogen bonds. The decrease in free hydroxyl groups was consistent with the peak shift at 3270 cm^−1^ detected by FTIR, suggesting that the phenolic hydroxyl groups of RES may consume free hydroxyl groups in two ways: directly forming hydrogen bonds with exposed peptide chain hydroxyl groups; and limiting hydroxyl accessibility through steric hindrance effects[30]. The enhancement of intramolecular hydrogen bonds was consistent with the increase in *β*-sheet content, as RES insertion into the hydrophobic core of *β*-sheets induced peptide chain conformational compaction, promoting intramolecular hydrogen bond formation[31].

2D-COS of FTIR spectra (Fig. 3E and 3F) reveals the molecular chronology of ultrasound-driven, RES-induced OVAf-LYZf fibril assembly. In the synchronous spectrum, prominent peaks at 1655 cm^−1^ and 1635 cm^−1^ indicate the concurrent disruption of *α*-helical structures and formation of amyloid *β*-sheets, with a strong positive cross-peak suggesting a direct, RES-mediated structural transition rather than a dissociative mechanism. The asynchronous spectrum, interpreted using Noda's rules, establishes the kinetic sequence: α-helix unfolding precedes *β*-sheet nucleation. A negative cross-peak at 1680 cm^−1^ emerges, signaling the formation of *β*-turn aggregates that compromise network homogeneity. LYZf contributes through electrostatic cross-linking, as evidenced by the synchronous correlation between its Amide II band (1520 cm^−1^) and OVAf *β*-sheets (1635 cm^−1^). Asynchronous analysis shows that RES aromatic stacking (1600 cm^−1^) occurs before *β*-sheet maturation, confirming its role as a molecular inducer.

### 3.3 Hydrocolloids properties and interface characteristics

**Fig. 4:**
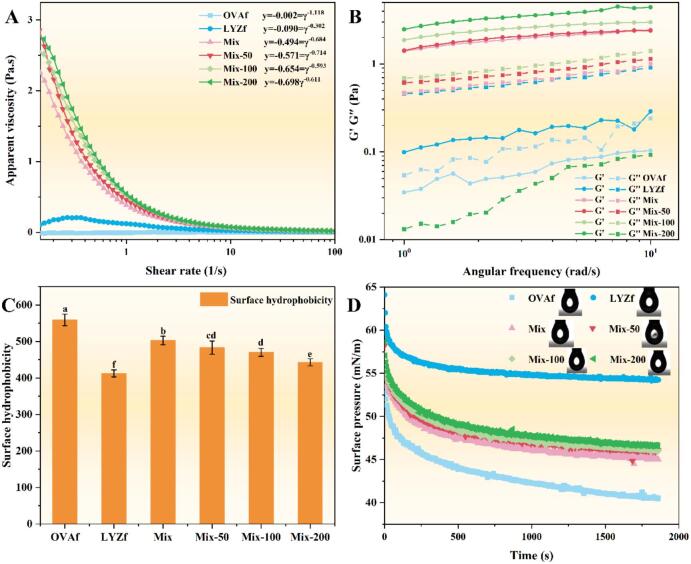
Rheological and interfacial properties of the nanofibril hydrocolloids: (A) Apparent viscosity; (B) Frequency sweep results (G' and G''); (C) Surface hydrophobicity; (D) Interfacial tension.

Rheological properties, including apparent viscosity and frequency sweep, are presented in Fig. 4A and 4B. OVAf and LYZf nanofibrils had not formed hydrocolloids, resulting in low apparent viscosity. After mixing the two fibrils, the apparent viscosity increased, with a significant elevation as RES concentration increased. The viscosity index *a* showed a marked upward trend, and all power-law indices *b* were less than 1, indicating non-Newtonian fluid behavior. At low concentrations, RES molecules may preferentially adsorb onto fibril surfaces, partially shielding electrostatic repulsion between fibrils and promoting short-range aggregation without forming a complete network structure, thus leading to only a slight increase in viscosity[32]. With increasing RES concentration, the molecules act as cross-linkers to bridge adjacent fibrils, forming a denser 3D network through polyphenol-protein interactions, which results in a significant rise in apparent viscosity. Ultrasound treatment may disrupt initially formed loose aggregates, forcing fibrils to disperse more uniformly and reorient under physical field effects, thereby synergizing with the chemical cross-linking of RES to enhance the homogeneity of the hydrocolloid structure[6].

The frequency sweep reveals a clear concentration-dependent reinforcement of the fibrillar network: the Mix-200 group achieves the highest G', progressively exceeding Mix-100, Mix-50, and the RES-free Mix group. This demonstrates that RES dose-dependently enhances inter-fibril crosslinking. All mixed systems exhibit G' > G'' across the frequency range of 0.1–10 rad/s. It indicates that the system exhibits obvious elastic (like solid) properties, confirming the formation of stable weak hydrocolloids. In contrast, OVAf alone forms a fragile network, while LYZf remains viscous-dominated. The near-parallel frequency dependence of G' for the Mix-200 group indicates a frequency-independent elastic network, whereas lower RES concentrations show more pronounced frequency dependence.

Surface hydrophobicity results are displayed in Fig. 4C. OVAf exhibited the highest surface hydrophobicity, while LYZf showed the lowest. For mixed systems, surface hydrophobicity decreased significantly with increasing RES concentration (*P* < 0.05). Electrostatic interactions between heterogeneous fibrils mask partial hydrophobic regions, leading to an overall decrease in hydrophobicity[33]. The hydrophobic aromatic rings of RES insert into the hydrophobic core of proteins, competitively occupying hydrophobic sites, while their phenolic hydroxyl groups remain on the fibril surface to form a hydrophilic layer[34]. Additionally, although RES-induced β-sheet restructuring locally increases order, it disrupts long-range hydrophobic interactions.

Changes in interfacial tension are shown in Fig. 4D. The OVAf group exhibited the lowest interfacial tension, which continued to decrease. In contrast, the LYZf group showed higher interfacial tension that tended to stabilize. Compared with pure OVAf, the interfacial tension of the mixed system increased. In the Mix-50 and Mix-100 groups, RES binds to nanofibrils through hydrophobic interactions, partially embedding into the fibril network and slightly adjusting its conformation without significantly altering the interfacial composition, resulting in overlapping interfacial tensions between the two groups [35], [36]. In the Mix-200 group, the high RES concentration may exceed the binding capacity of nanofibrils, with some free molecules occupying the interface or inducing fibril aggregation to form a looser interfacial structure, leading to a slightly higher interfacial tension than the low-concentration groups[37].

### 3.4 Emulsion-hydrocolloid composite properties and microstructure

**Fig. 5:**
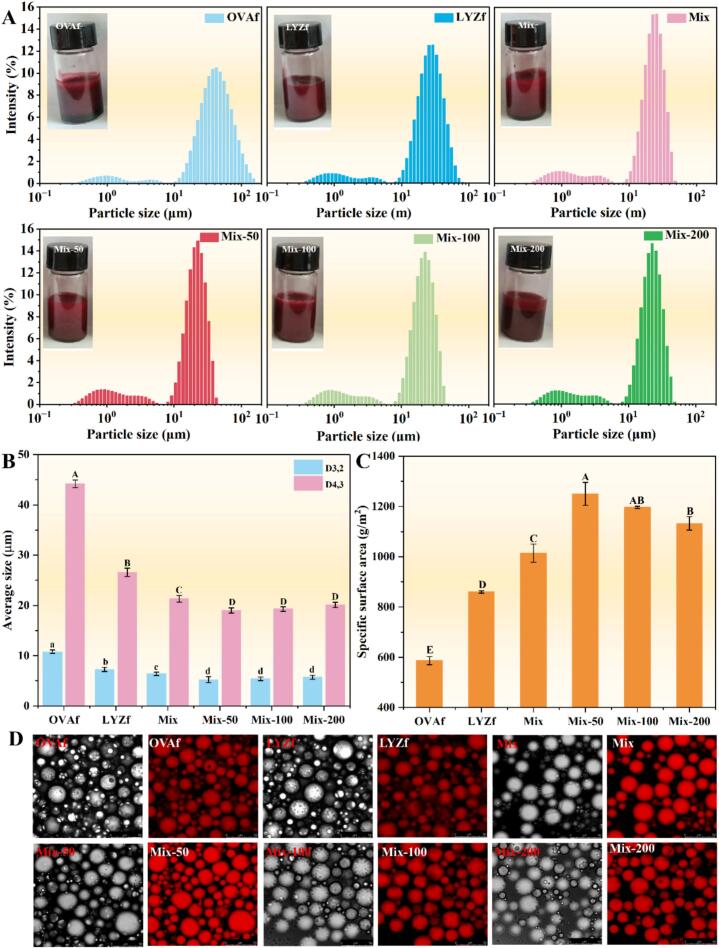
Characterization of C3G-loaded emulsions stabilized by the nanofibril hydrocolloids. (A) Visual appearance and particle size distribution of the emulsion hydrocolloid; (B) Average particle size (D_3,2_ and D_4,3_); (C) Specific surface area; (D) Optical and CLSM images of emulsion droplets. Different lowercase letters above the bars indicate significant differences (*P* < 0.05).

Droplet size distribution and morphological characteristics of the emulsions are shown in Fig. 5A. All emulsions exhibited bimodal size distributions, with peaks concentrated at 0.86–1.01 μm and 21.9–41.9 μm. OVAf-stabilized emulsions showed the largest main peak, while the main peak of emulsions stabilized by the mixed group decreased with increasing RES concentration. The D_4,3_ was calculated, and the results are presented in Fig. 5B. The OVAf group had the largest D_4,3_, followed by the LYZf group. The mixed system showed a further decrease in average droplet size, though no significant difference was observed (*P* > 0.05). OVAf easily forms a loose network through hydrophobic interactions to encapsulate oil droplets, and its low rigidity results in insufficient mechanical strength of the interfacial film. In contrast, LYZf reduce steric hindrance to form a dense interfacial layer, while enhancing stability through electrostatic interactions. The mixed system exhibited superior stability, benefiting from the charge complementarity of the co-assembled OVAf-LYZf[13]. RES embedded into the hydrophobic core of fibrils to enhance structural stability, and its phenolic hydroxyl groups formed hydrogen bond networks with surface groups of fibrils to improve interfacial continuity, additionally exerting a synergistic antioxidant effect with C3G[13].

The specific surface area (SSA) of the emulsions was further determined, with results displayed in Fig. 5C. The OVAf group had the smallest SSA, while the LYZf group showed a significantly higher SSA than OVAf (*P* < 0.05). Emulsions in the mixed groups and RES-added groups exhibited significantly higher SSA than both OVAf and LYZf groups (*P* < 0.05). However, the SSA decreased significantly with increasing RES concentration (*P* < 0.05). The slight increase in droplet size combined with a significant decrease in SSA indicates that RES-induced fibrous hydrocolloids at high concentrations form non-adsorptive aggregates during dispersion, reducing interfacial coverage[38]. CLSM images (Fig. 5D) showed that fat droplets in all emulsions were uniformly dispersed with a homogeneous state, confirming that the nanofiber composite system can effectively stabilize emulsions.

### 3.5 Fiber hydrocolloid modulates the emulsion- hydrocolloid rheology properties

**Fig. 6:**
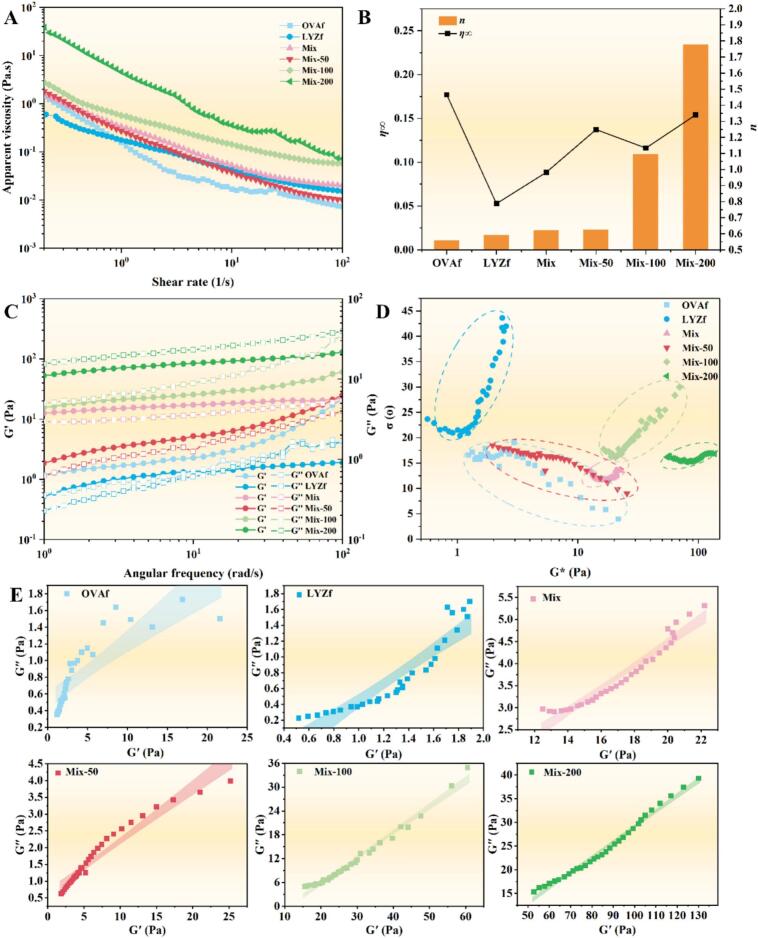
Rheological properties of the C3G-loaded emulsion hydrocolloid: (A) Apparent viscosity; (B) Infinite shear viscosity and shear-thinning index; (C) Frequency sweep results (G' and G''); (D) van Gurp-Palmen plot (phase angle δ vs. lgG*); (E) Cole-Cole plot (G'' vs. G').

The apparent viscosity of all emulsions decreased significantly with increasing shear rate (Fig. 6A), indicating shear-thinning fluid behavior. With the addition of RES, the viscosity of emulsion hydrocolloids gradually increased, with the Mix-200 group exhibiting the highest viscosity. RES facilitates inhibiting oil droplet sedimentation/coalescence and improving emulsion storage stability. Fitting with the Cross-model revealed an increase in *n*, indicating weakened shear thinning (Fig. 6B). Particle cross-linking of the hydrocolloid at the interface resulted in a denser emulsion hydrocolloid network structure, increasing the difficulty of structural destruction under shear and leading to a smoother viscosity change during processing[10]. OVAf exhibited the highest *η*_∞_, while LYZf showed the lowest (Fig. 6B). With the co-assembly of blended fibrils and the action of RES, *η*_∞_ gradually increased, indicating enhanced cross-linking degree of blended fibrils, increased internal friction under high shear, and elevated lower limit of fluidity, avoiding excessive thinning during processing.

Frequency sweep results are displayed in Fig. 6C. Consistent with viscosity changes, G' increased significantly with hydrocolloid formation. The Mix-200 group showed the highest G' and G'', with moduli tending to plateau as frequency increased. This indicates that this group had the densest hydrocolloid network and the strongest structural rigidity. The network was resistant to irreversible damage when responding to external dynamic disturbances, enabling stable encapsulation of oil droplets and preventing coalescence. The Van Gurp-Palmen plot is a “fingerprint” in rheology (Fig. 6D) [39]. For the LYZf group, the phase angle increased significantly with lgG*, showing the largest phase angle variation (up to 41.97°), indicating a viscosity-dominated system with a loose amyloid fibril network and weak binding capacity to oil droplets. In contrast, the phase angle of OVAf gradually decreased with lgG*, a trend also observed in the Mix-50 group, suggesting that the elastic contribution increased with enhanced network strength and fibril cross-linking. After mixing OVAf and LYZf, the phase angle variation range became gentle and narrowed, reflecting that interactions between blended fibrils led to more uniform viscoelastic distribution and improved network homogeneity[35]. The Mix-100 group showed a significant increase in phase angle, presumably due to the transitional state of fibril cross-linking regulation by RES at this concentration. The phase angle of the Mix-200 group became further gentle, indicating that high-concentration RES fully strengthened the cross-linked network of blended fibrils, returning the system to an elastic-dominated, structurally stable state, well-suited for long-term emulsion stability requirements.

The Cole-Cole plot systematically reveals the evolution of network homogeneity from OVAf to the Mix-200 group (Fig. 6E). OVAf exhibited a broad distribution with a wide confidence interval, corresponding to a multi-relaxation mode, indicating a loose and heterogeneous network structure prone to local oil droplet coalescence. Although LYZf showed a narrowed confidence interval, the intercept remained below 1.0 Pa, reflecting weak initial network strength and insufficient binding capacity of hydrocolloid fibrils to oil droplets. In contrast, the mixed system was gradually optimized with RES addition: the confidence interval further narrowed, and network homogeneity and initial strength improved synchronously, resulting in more uniform encapsulation of oil droplets[40]. The intercept of the Mix-200 group jumped to over 10 Pa, forming a straight line without arc segments, demonstrating that high-proportion RES transformed the multi-mode network into a highly homogeneous and dense structure. The single-relaxation structure provides uniform and stable binding force to oil droplets, representing the optimal network state for ensuring long-term dispersion stability of C3G emulsions.

### 3.6 The stability of emulsion- hydrocolloid and aqueous phase C3G

**Fig. 7:**
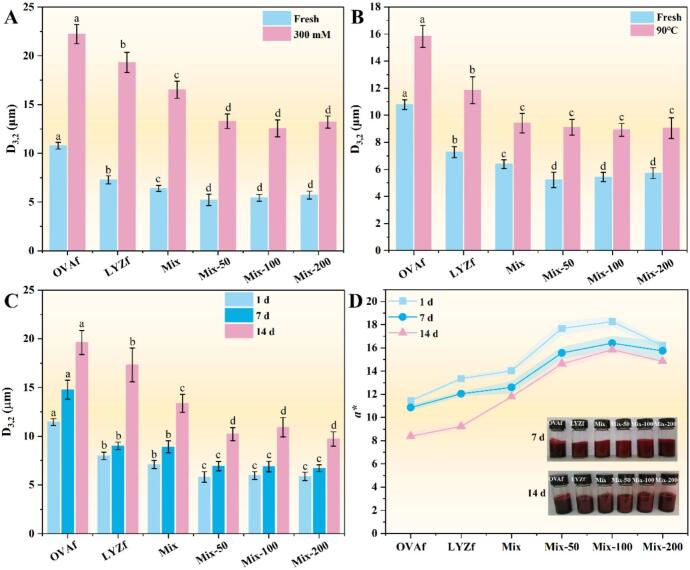
Stability evaluation of the C3G-loaded emulsion hydrocolloid: stability under (A) ionic strength (300 mM NaCl), (B) heating (90 °C for 30 min), and (C) long-term storage (14 days). (D) The a* value of C3G in the emulsion during storage. Different lowercase letters above the bars indicate significant differences (*P* < 0.05).

Changes in the average particle size of emulsions under conditions of 300 mM NaCl, heating at 90°C for 30 min, and during storage are shown in Fig. 7A–C. NaCl exerted the most significant effect on the particle size of all groups. After heating for 30 min, the oil droplet size increased remarkably, and a significant increase was observed during the storage period (*P* < 0.05). The OVAf-stabilized emulsion exhibited the largest particle size, while the mixed emulsion had a smaller particle size (*P* < 0.05). However, no significant change in particle size was observed in RES-added emulsions (*P* > 0.05). The OVAf-stabilized emulsion consistently maintained the largest particle size. Hydrophobic interactions between OVAf fibrils strengthened over time, forming a denser network structure, while hydrophobic fragments generated by partial fibril degradation promoted oil droplet coalescence. In contrast, the 3D network formed by the complementary OVAf and LYZf fibrils effectively hindered oil droplet migration, and the charge neutralization effect reduced environmental sensitivity. The introduction of RES maintained the emulsion particle size stability during storage. Its antioxidant properties prevented oxidative cross-linking of interfacial proteins, and the hydrogen bond network formed with fibrils enhanced interfacial mechanical strength, inhibiting oil droplet coalescence[38].

Color changes of emulsions were determined to reflect the stability of C3G in the aqueous phase, with results displayed in Fig. 7D. After initial storage, a* values of all systems ranged from 11.45 to 18.25, exhibiting good consistency in red color, indicating uniform initial dispersion of C3G and a stable emulsion state. After 7 days of storage, the a* values of OVAf and LYZf groups decreased more significantly, while the rate of decrease was remarkably narrowed in the Mix series, especially Mix-100 and Mix-200. The preliminary cross-linking of blended fibrils in the Mix system improved network compactness, temporarily delaying the degradation process. After 14 days of storage, the a* values of OVAf and LYZf groups continued to decrease significantly. For the Mix series, with increasing RES concentration, the rate of a* decrease gradually diminished, and the emulsions retained a uniform deep red color, as shown in photographic images. The homogeneous and dense hydrocolloid network in the Mix-200 system not only effectively inhibited oil droplet coalescence over the long term but also stably encapsulated C3G.

### 3.7 *In vitro* digestive behavior

**Fig. 8:**
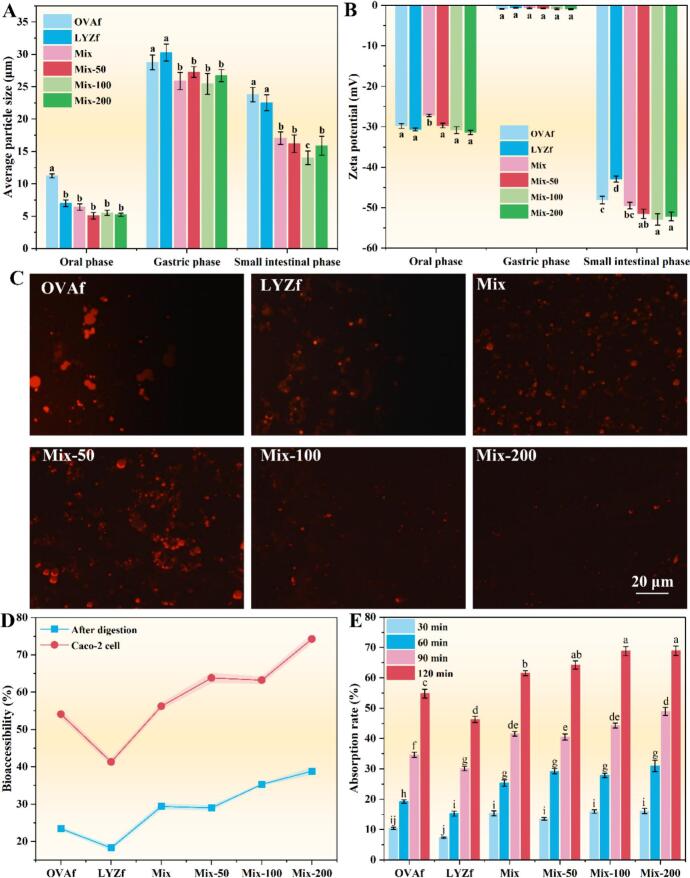
*In vitro* digestion of the C3G-loaded emulsion: changes in (A) particle size, (B) zeta potential, and (C) CLSM microstructure during digestion; (D) Bioaccessibility of C3G and its absorption rate (E) by Caco-2 cell monolayers over 120 min. Different lowercase letters above the bars indicate significant differences (*P* < 0.05).

**Fig. 9:**
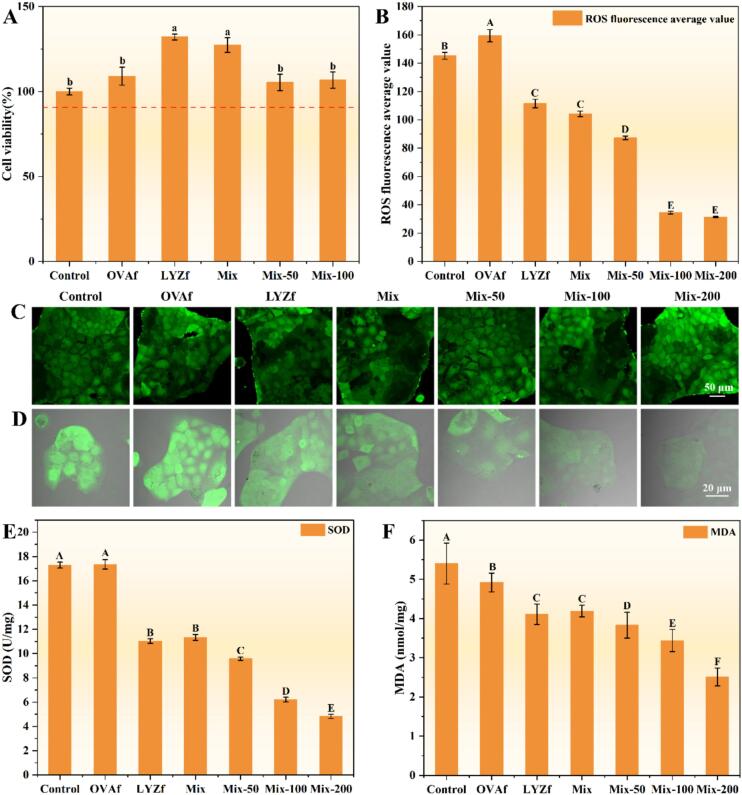
Cytoprotective effects of the digestive micelles from the emulsion on Caco-2 cells under oxidative stress (100 μM H_2_O_2_ for 4 h): (A) Cell viability; (B) Average intracellular ROS fluorescence intensity; (C) Live/Dead cell staining (calcein-AM/PI); (D) ROS fluorescence imaging; (E) SOD activity; (F) MDA content. Different lowercase letters above the bars indicate significant differences (*P* < 0.05).

An in vitro simulation was used to evaluate the digestive behavior of the emulsions, with changes in particle size, ζ-potential, and microstructure presented in Fig. 8A, 8B, and 8C. During the oral phase, the OVAf group displayed a significantly larger mean particle size compared to the other groups (*P* < 0.05), among which no significant differences were detected (*P* > 0.05). The Mix group showed the lowest absolute ζ-potential (*P* < 0.05), while the remaining groups exhibited comparable values (*P* > 0.05). This result indicates that effective charge neutralization occurred between OVAf (negatively charged) and LYZf (positively charged), leading to the formation of a more charge-neutral composite fiber network. The large aggregates observed in the OVAf emulsion were attributed to the partial dissociation of long fibrils by salivary α-amylase, combined with strong hydrophobic interactions and electrostatic complexation between negatively charged fibrils and salivary mucin. In contrast, partial charge neutralization between OVAf and LYZf, along with hydrogen bonding mediated by RES's phenolic hydroxyl groups, contributed to modified interfacial charge distribution in the mixed and RES-enhanced systems. In the gastric phase, all emulsions showed a significant increase in particle size (*P* < 0.05). The OVAf and LYZf groups formed larger aggregates than the RES-containing groups (*P* < 0.05), though no significant difference was observed between these two (*P* > 0.05). The remaining groups did not differ significantly in size (*P* > 0.05). No notable differences in ζ-potential were detected across groups (*P* > 0.05). Acid-induced unfolding of protein fibrils exposed hydrophobic regions, thereby promoting aggregation. Concurrently, pepsin cleavage generated aggregation-prone peptides[36]. During the intestinal phase, emulsions underwent decomposition, reflected in a significant decrease in average particle size compared to the gastric phase (*P* < 0.05). The OVAf and LYZf groups retained the largest particle sizes, with no significant difference between them (*P* > 0.05). The Mix, Mix-50, and Mix-200 groups showed comparable sizes (*P* > 0.05), while the Mix-100 group had the smallest. RES-added groups exhibited the highest absolute ζ-potential values, with no significant differences among them (*P* < 0.05), whereas the LYZf group showed the lowest. The OVAf and Mix groups shared similar ζ-potential values (*P* > 0.05). CLSM images (Fig. 8C, labeled with rhodamine B) revealed considerable microstructural changes after intestinal digestion. Most proteins detached from droplet surfaces and formed aggregates, although RES- supplemented emulsions displayed more complete digestion and reduced aggregation. The rise in pH during the intestinal phase prompted charge reorganization, attenuating the positive charge of LYZf and mitigating electrostatic aggregation. Pancreatic lipase disrupted the oil–water interface, leading to droplet breakup and partial degradation of the fibril network. RES-fibril complexes modulated trypsin cleavage accessibility, facilitating the formation of degradable intermediates. Additionally, RES's antioxidant capacity helped maintain appropriate protein unfolding, and its synergistic interaction with bile salts enhanced mixed micelle formation[41] (see Fig. 9).

### 3.8 Transmembrane transport of C3G in micelles

The transmembrane transport efficiency of C3G-loaded emulsions was assessed using a Caco-2 cell monolayer model (Fig. 8D and 8E). The LYZf-stabilized emulsion exhibited the lowest C3G bioaccessibility following digestion. No significant difference was detected between the OVAf and Mix groups (*P* > 0.05). The Mix-50 and Mix-100 groups showed significantly higher bioaccessibility than the fibril-only groups but lower than the Mix-200 group (*P* < 0.05). After cell culture, the LYZf group still exhibited the lowest bioaccessibility, followed by OVAf. The Mix and Mix-50 groups did not differ significantly (*P* > 0.05), whereas the Mix-200 group outperformed all others (*P* < 0.05). These findings indicate that RES-enhanced formulations facilitated C3G absorption and conversion.

The limited bioaccessibility of the LYZf emulsion was attributed to its structural characteristics: densely packed aggregates formed by short fibrils during gastric digestion (Fig. 8C) restricted pancreatin accessibility, resulting in incomplete C3G release. Moreover, the diminished positive surface charge of lysozyme fibrils under intestinal pH conditions reduced electrostatic interaction with the anionic intestinal mucus, thereby shortening mucoadhesion and retention[42]. In contrast, the Mix-200 group achieved optimal bioaccessibility (*P* < 0.05), owing to the moderately open architecture of RES–fibril complexes in the GI tract, which enhanced enzyme accessibility. Additionally, 200 μM RES promoted the incorporation of hydrophobic C3G derivatives into bile salt micelles via compositional modulation.

C3G uptake profiles in Caco-2 cells incubated with micelles (Fig. 8E) further substantiated the emulsion functionality. At 60 min, all groups showed absorption rates below 50%, with LYZf being the lowest, followed by OVAf. Absorption increased significantly upon combining OVAf with LYZf and with elevating RES concentration (*P* < 0.05). By 120 min, LYZf and OVAf remained the least absorbed, whereas Mix-100 and Mix-200 attained the highest uptake. Initially, the cell membrane impeded permeation of hydrophilic C3G. Over time, RES facilitated *π–π* stacking with C3G[11], increasing its lipophilicity and promoting passive diffusion across the membrane.

### 3.9 Antioxidant stress resistance

Cell viability analysis (Fig. 9A) revealed that all treatment groups exhibited viability exceeding 95%, with significantly higher values than the blank group, indicating no cytotoxic effects. Live/dead staining using AM/PI further confirmed that all groups maintained high cell viability without detectable cell death (Fig. 9C). To evaluate oxidative stress mitigation, Caco-2 cells were pre-treated with H_2_O_2_ and subsequently co-cultured with micelles. Intracellular ROS were labeled with DCFH-DA and visualized (Fig. 9D). The control and OVAf groups showed the highest ROS fluorescence intensity. The fluorescence signal decreased with the incorporation of OVAf and LYZf, and further declined with increasing RES concentration. Quantitative analysis of ROS fluorescence (Fig. 9B) confirmed that the control and OVAf groups had the highest ROS levels. No significant difference was observed between LYZf and Mix groups (*P* > 0.05), while a dose-dependent reduction in ROS occurred with higher RES content (*P* < 0.05), indicating a synergistic antioxidant effect between RES and C3G.

Oxidative stress parameters were further assessed by measuring SOD activity and MDA content (Fig. 9E and 9F). The control and OVAf groups exhibited the highest SOD activity. A significant decrease was observed in the LYZf and Mix groups (*P* < 0.05), with a further reduction as RES concentration increased. A similar trend was observed for MDA content, confirming mitigation of lipid peroxidation. A clear dose–response relationship was identified between RES concentration and ROS scavenging efficacy. The RES-C3G complex acts as an electron donor, directly neutralizing free radicals and modulating mitochondrial electron transport chain activity to suppress ROS generation at the source[43]. The elevated SOD activity in the blank and OVAf groups reflects a compensatory response to severe oxidative stress. In contrast, the moderate reduction in SOD activity in RES-treated groups, particularly Mix-200, suggests better maintenance of redox homeostasis and reduced dependency on enzymatic antioxidants. Additionally, RES interacts with unsaturated membrane lipids via π-alkyl stacking, effectively inhibiting lipid peroxidation cascades[22].

## 4 Conclusion

This study successfully developed a novel protein-based delivery system for C3G via a synergistic strategy integrating low-frequency ultrasound and RES induction. The core innovation lies in the elucidated mechanism where ultrasound provides the physical energy for fibril dispersion and alignment, while RES acts as a molecular bridge, facilitating fibril cross-linking into a robust three-dimensional network through non-covalent interactions and *β*-sheet rearrangement. The resulting hydrogel effectively stabilized C3G-loaded emulsions, which exhibited uniform droplet distribution, enhanced stability, and improved bioaccessibility and cellular uptake in Caco-2 models. This work not only provides a promising approach for stabilizing hydrophilic bioactive compounds in functional foods, but also offers fundamental insights into the ultrasonic modulation of protein-based nanostructures. While the current study validates preliminary safety in cellular models, future work should include comprehensive in vivo assessments of bioavailability and biosafety. Further engineering of the hydrogel to incorporate environmental responsiveness represents a promising avenue for developing intelligent targeted delivery systems in the pharmaceutical and nutraceutical industries.

## CRediT authorship contribution statement

**Xuke Han:** Writing – original draft, Methodology, Formal analysis, Conceptualization. **Chan Zhu:** Validation, Investigation, Data curation. **Yifeng Shen:** Resources, Methodology. **Minmin Ai:** Supervision, Resources. **Lue Ha:** Supervision, Resources.

## Declaration of competing interest

The authors declare that they have no known competing financial interests or personal relationships that could have appeared to influence the work reported in this paper.
