# Supplementary material for: Ultrasonic driven resveratrol induced egg-derived amyloid-like fibrils hydrocolloids for stabilizing aqueous cyanidin-3-O-glucoside
Source: Ultrason Sonochem. 2026 Feb 8;127:107767. doi: 10.1016/j.ultsonch.2026.107767 (PMC12925334; doi:10.1016/j.ultsonch.2026.107767)
Supplement: Supplementary Data 1 [file mmc1.docx]

**Ultrasonic driven resveratrol induced egg-derived amyloid-like fibrils hydrocolloids for stabilizing** **aqueous cyanidin-3-O-glucoside**

Xuke Han^1,2^, Chan Zhu^3^, Yifeng Shen^4^, Minmin Ai^5^, Lue Ha^1,2*^

^1^Key Laboratory of Acupuncture and Medicine in Shaanxi Province, College of Acupuncture & Tuina, Shaanxi University of Chinese Medicine, Xianyang 712046, China

^2^Shaanxi TCM Diagnosis and Treatment Technology and Equipment R&D Collaborative Innovation Center, Shaanxi University of Chinese Medicine, Xianyang 712046, China

^3^Key Laboratory of Birth Defects and Related Gynecological Diseases, Traditional Chinese Medicine Department, West China Second Hospital of Sichuan University, Chengdu 610021, China

^4^TCM Regulating Metabolic Diseases Key Laboratory of Sichuan Province, Hospital of Chengdu University of Traditional Chinese Medicine, Chengdu 610032, China

^5^College of Food Science, South China Agricultural University, Guangzhou 510642, China

***Correspondence:** Lue Ha: College of Acupuncture & Tuina, Shaanxi University of Chinese Medicine, Email: [halue@126.com](mailto:halue@126.com)

**Methods for calculating diffusion coefficient:**

The adsorption process involves diffusion, permeation, and rearrangement (complexation). In the initial adsorption stage, the surface pressure (π) related to adsorption time (t) is estimated using the following formula:

$$\pi={2C_{0}KT(D_{t}/3.14)}^{1/2}$$

Where *C_0_* is the bulk concentration, *K* is Boltzmann’s constant, *T* is the absolute temperature, *D* is the diffusion coefficient, and t is the adsorption time.

If the adsorption process is diffusion-controlled, $\pi$ versus *t^1/2^* will show linearity, and the slope represents the diffusion rate (*K_diff_*) for composite particles. The penetration rate and rearrangement rate are analyzed using the equation proposed by Graham and Philips:

$$ln\left[ \frac{(\pi_{f}-\pi_{t})}{(\pi_{f}-\pi_{0})} \right]=-k_{i}t$$

**Results:**

**1. The function of ultrasound**

This study intuitively elucidates the regulatory effect of ultrasonication on the microstructure of OVAf and LYZf, and the RES mixed fibrils via atomic force microscopy (AFM). Before ultrasonication, the samples were characterized by the cluster-like accumulation of protein aggregates and colloidal particles, exhibiting poor dispersibility and extremely uneven distribution; the surface height difference reached the scale of tens to hundreds of nanometers, showing distinct microscopic morphological heterogeneity (Fig. S1). After ultrasonication, the samples were successfully transformed into continuous and uniform fibrous assemblies (Fig. 1). The aggregated structures were effectively depolymerized under the synergistic effect of ultrasonic mechanical shearing and cavitation effect, enabling homogeneous co-assembly of different fibrils components at the molecular level. Consequently, the surface height difference was reduced to the nanoscale, and the flatness and uniformity of the microstructure were significantly improved.

**
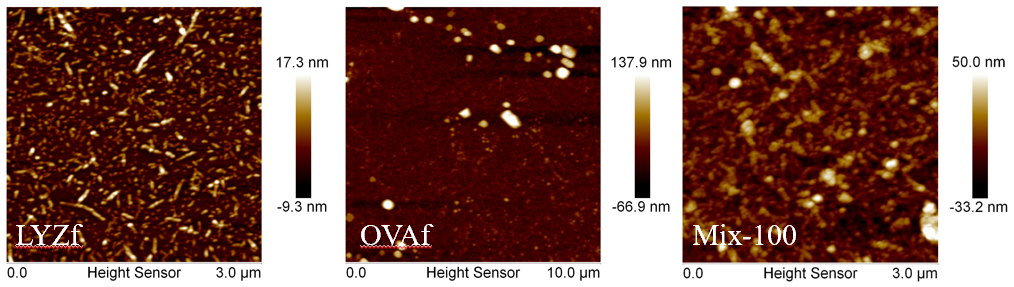
**

Fig. S1 The AFM results of LYZf, OVAs and Mix-100 group before ultrasound.

**2.The diffusion coefficient**

The diffusion coefficients across different fibril systems revealed distinct characteristics: LYZf exhibited the highest value, while OVAf showed the lowest. A significant increase (*P* < 0.05) was observed upon mixing OVAf with LYZf and with elevating RES concentrations (Fig. S2). This trend is mechanistically explained by the shorter length and lower entanglement density of LYZf, which facilitate mobility, contrasted by the extensive length and strong intermolecular interactions of OVAf that restrict diffusion. The OVAf/LYZf mixture reduced the system’s overall viscosity, and the incorporation of RES further weakened inter-fibril hydrogen bonding and hydrophobic interactions, collectively enhancing molecular mobility. Regarding emulsification, the low interfacial tension of OVAf is conducive to forming small droplets, though its slow diffusion may hinder the emulsification process. Conversely, LYZf’s high diffusion coefficient favors rapid adsorption, yet its high interfacial tension can compromise droplet stability. The modulating effect of RES on the mixed system appears to strike a balance between rapid interfacial adsorption (kinetics) and effective interfacial structuring (thermodynamics), thereby potentially enhancing both the initial uniformity and the long-term stability of the resulting emulsions.


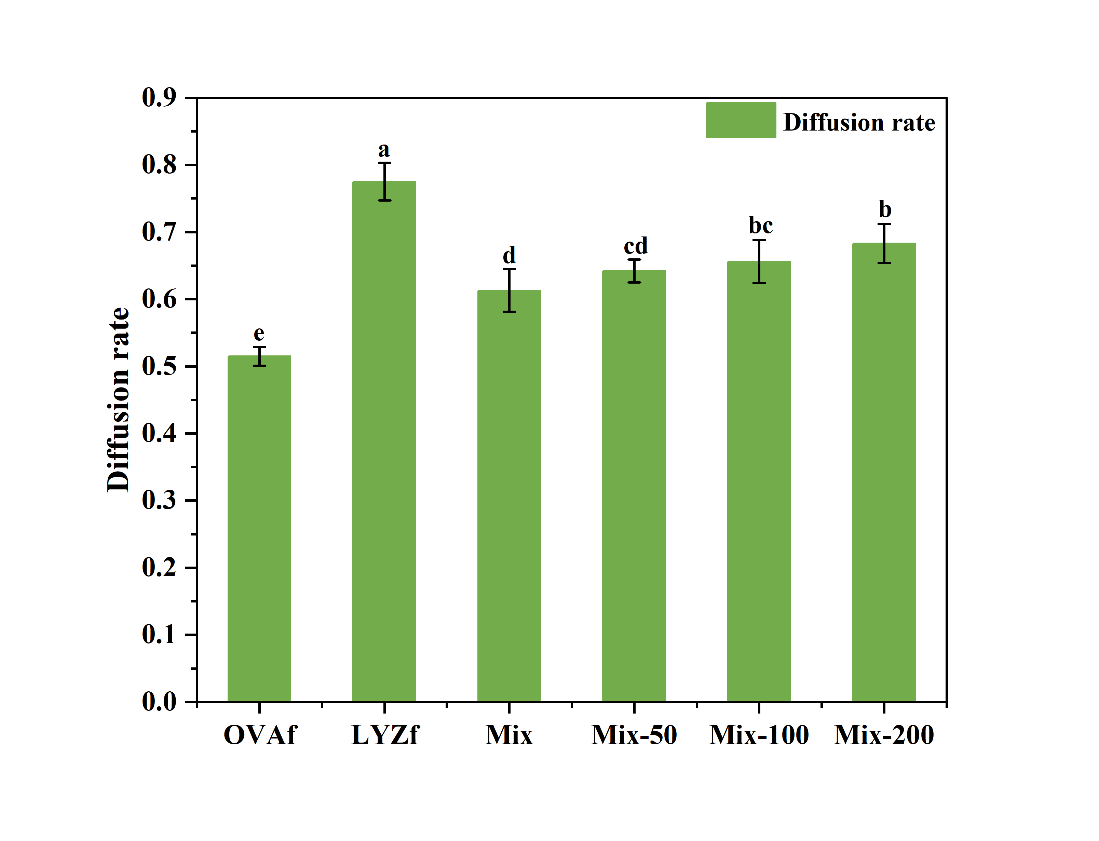


Fig. S2 The diffusion coefficient of different samples. Different lowercase letters above the bars indicate significant differences (*P* < 0.05).
